# Estrogen Status Influences Whole-Body Vibration Training-Induced Improvements on Muscle Mass and Strength in Female Ovariectomized Mice

**DOI:** 10.7150/ijms.97770

**Published:** 2024-08-12

**Authors:** Xiangyang Tian, Cong Li, Tao Li, Fangfang Yu, Rengfei Shi

**Affiliations:** School of Exercise and Health, Shanghai University of Sport, Shanghai, China.

**Keywords:** estrogen, whole-body vibration training, muscle mass and strength, body composition, Akt-mTOR, FoxO1

## Abstract

Estradiol (E2) deficiency arising from menopause is closely related to changes in body composition and declines of muscle mass and strength in elderly women. Whole-body vibration training (WBV) is an emerging approach expected to improve muscle mass and strength of older person, but the underlying mechanisms remain unclear. The balance between protein synthesis and degradation is a determining factor for muscle mass and strength, which is regulated by Akt-mTOR and FoxO1 signal pathway, respectively. In the present study, we firstly determined whether the effects of WBV on muscle mass and strength in ovariectomized female mice was affected by estrogen level, then investigated whether this was associated with Akt-mTOR and FoxO1 signal pathways. We found that (1) WBV, E2 supplementation (E) and WBV combined with E2 supplementation (WBV+E) significantly increased serum estradiol content, quadriceps muscle mass and grip strength in ovariectomized mice, accompanied with alterations of body composition (reducing fat content, increasing lean body mass and lean percent), furthermore, the altered degrees of these indicators by WBV+E were greater than WBV alone; (2) WBV, E and WBV+E remarkably increased the activities of Akt and mTOR and decreased FoxO1 activity, and the changed degrees by WBV+E were greater than WBV alone; (3) Pearson correlation coefficient revealed that serum estradiol content was positively correlated with Akt and mTOR activities, while inversely associated with FoxO1 activity. We concluded that WBV could significantly increase muscle mass and strength in ovariectomized mice, which might achieve through activating Akt-mTOR and suppressing FoxO1 signal pathways, and the improving effect of WBV on muscle mass and strength was better when in the presence of estrogen.

## Introduction

As with advancing aging, older individuals undergo muscle mass and strength decrease progressively, which was also designated as sarcopenia 1, 2. This reduction of muscle mass and strength causes several adverse outcomes, such as falls and fractures 3, cognitive impairment 4 and pulmonary insufficiency 5, all of which negatively affect the independence and the quality of life of older individuals 3. With the rapid growth of elderly population, estimated by World Health Organization that the global population of individuals aged over 60 years will nearly double from 12% to 22% between 2015 and 2050, increasing muscle mass and function of elderly individuals is an urgent issue to be solved, especially for female who have a longer life expectancy but with a higher prevalence of low muscle mass, and experience faster muscle strength declines after menopause 6.

Physical activity insufficiency is considered as an important factor leading to the decrease of muscle mass and strength. A great body of evidence from human and animals demonstrated that exercise training, especially resistance training could effectively prevent or delay the occurrence and development of sarcopenia in older individuals 7-9. However, the benefits of resistance training on muscle mass and strength are elicited by its high mechanical loading (up to 70%-90% 1RM), most elderly persons are unable or unwilling to comply with high-intensity exercise regimens 10. Whole-body vibration training (WBV) is an emerging training method that utilizes vibration platform with different vibration frequency and amplitudes in different positions to induce muscle contraction passively, and widely adopted by older adults because of its safety and effectiveness on muscle mass and strength, for instance, 12-week WBV remarkably improved skeletal muscle mass index, physical fitness (including standing on foot, shoulder-arm flexibility, 8-ft up and go test, hand grip strength and five repeated sit-to stand tests) and quality of life of sarcopenic older people 11; A meta-analysis of randomized controlled trials also indicated that WBV could significantly increase skeletal muscle mass, muscle strength and physical performance in older adults with sarcopenia 12, thus WBV may be an alternative to resistance training for improving muscle mass and function in older adults with sarcopenia.

Estrogen, in addition to its effects on female reproductive system development or maintenance, has various physiological functions in non-reproductive tissue such as cardiovascular system, liver and skeletal muscle 13. In mammalian, there are three major bioactive estrogens: estrone (E1), estradiol (E2) and estriol (E3), and E2 is the most potent estrogen that primarily secreted by ovaries and released into blood. Ovariectomy is a commonly used approach to simulate menopause transition in female and widely applied in researches about the effects of estrogen deficiency caused by menopause on peripheral tissues or organs. For example, OVX resulted in the increase of hepatic triglyceride content 14, induced arterial senescence and the development of atherosclerosis 15. Multiple modalities exercise training, such as treadmill exercise and voluntary wheel running could significantly reduce liver fat accumulation or improve vascular endothelial function, but the benefits of exercise on liver or endothelial function was affected by estrogen status, as the improving effects of exercise on liver or vascular endothelial function were better when in the presence of estrogen 16-18. For skeletal muscle, estrogen deficiency also has been demonstrated to be associated with decreased muscle mass and strength 19, 20, and exogenous E2 administration could improve age-associated muscle mass and strength 21, 22, but whether the effects of WBV on muscle mass and strength were influenced by estrogen status needs to be clarified.

The balance between protein synthesis and degradation is an important factor affecting muscle mass and strength. Mammalian target of rapamycin (mTOR) is the primary positive regulator of protein synthesis, and forkhead box O1 (FoxO1) is the important regulator of protein degradation in skeletal muscle through regulating muscle-specific ubiquitin E3 ligases: muscle atrophy F-box (MAFbx) or muscle ring-finger protein 1 (MuRF1). The activities of mTOR and FoxO1 are regulated by phosphorylation, mTOR phosphorylation induces the downstream translation initiation and promotes protein synthesis, whereas FoxO1 phosphorylation results in its location in cytoplasm, then suppressing MAFbx and MuRF1 expression and protein degradation. The phosphorylation of mTOR and FoxO1 are regulated by several kinases, such as protein kinase B (PKB/Akt). Evidence from *in vivo* and *in vitro* studies demonstrated that several molecules via activating Akt-mediated mTOR and inhibiting FoxO1 signaling pathway 23-25 promoted muscle hypertrophy or prevented muscle atrophy. Estradiol could also regulate mTOR and FoxO1 activities. 17β-estradiol promotes the proliferation of pancreatic β-cell and insulin secretion via regulating FoxO1 and Akt-mTOR pathway 26, 27, respectively. The improvement of 17β-estradiol on insulin sensitivity and suppression on gluconeogenesis were fulfilled through Akt-FoxO1 pathway 28.

In the present study, we intended to firstly determine the effects of whole-body vibration training on muscle mass and strength in ovariectomized mice and whether the effects of WBV was affected by estrogen level, then explored whether WBV increased muscle mass and strength via Akt-mediated mTOR and FoxO1 pathways.

## Materials and Methods

### Animals and groups

Forty female C57BL/6 mice (10 weeks old) were housed in temperate- (22℃) and humidity- (50%) controlled room with 12 h light: dark cycle and free access to food and water. All animal treatments were in accordance with the guidelines, and experiment procedures were authorized by the Ethics Committee of Shanghai University of Sport (102772022DW032). After experiencing bilateral ovariectomy surgeries (OVX), mice were randomly assigned into four groups: OVX, OVX plus estradiol supplementation (OVX+E), OVX plus whole-body vibration training (OVX+W), OVX plus estradiol supplementation and whole-body vibration training (OVX+W+E) following recovery for two weeks.

### Ovariectomy and 17-β estradiol supplementation

All mice underwent ovariectomy under anesthesia, and the experiment procedures were performed under sterile conditions. Briefly, mice were anesthetized with intraperitoneal injection of 3% pentobarbital sodium, then fixed onto anatomical plate in a stoop-over position. A 1cm incision was cut from both sides of spines after hair on the beck was removed off, then the ovaries were excised. Without obvious bleeding in mice, the wound was sutured. After 2 weeks recovery, vaginal smear was performed to determine whether ovariectomy was successfully performed.

After 2 weeks recovery from ovariectomy, mice in OVX+E and OVX+W+E groups were administrated estradiol via subcutaneously implantation with 17-β estradiol releasing pallets (0.36 mg/pellet, evenly releases for 90 days, Innovative Research of America Company) prior to exercise intervention. Mice in OVX and OVX+W groups underwent the surgery but no pallets were embedded.

### Whole-body vibration training protocol

Mice in OVX+W and OVX+W+E groups experienced whole-body vibration training on a vertically oscillating platform (LD-20BL, Longdate, Guangzhou, China). The vibration stimulus was applied on a vertical amplitude of 3 mm and a vibration frequency of 30 Hz with an acceleration of 5.4 g lasting for 30 min, 5 d/week for 8 weeks, and the vibration process was continuous without interruption or rest. During vibration, mice were housed in 1 of 16 compartments of an acrylic cage fixed on the top of the platform. Mice in OVX and OVX+E groups were placed in an identical apparatus for the same duration but didn't underwent any vibration stimulus.

### Detection of body composition by Echo MRI-100 body composition analyzer

The body composition of all mice was determined by Echo MRI Body Composition Analyzer (Echo Medical Systems, Houston, USA) without anaesthetization before ovariectomy and after exercise or estrogen administration. Briefly, mice were placed in a tube for fixation and put into apparatus for scanning according to manufacturer's instructions, then fat content, lean mass of each mouse were calculated.

### Measurement of grip strength and muscle mass

The forelimb grip strength was measured by grip-strength meter (YLS-13A, Jinan Yiyan Technology Co., Ltd. Jinan, China) before and after ovariectomy and exercise intervention. In brief, all mice were placed in the testing room for 30 min to acclimate, then each mouse was placed over the top of the grid of the grip-strength meter. After grasping the grid with four paws, the mouse was pulled backwards along the axis of grip strength meter. The speed should be slow enough to let the mice to resist against the pulling force, then the value would be displayed on the screen of the grip-strength meter after the mice released from the grid. Each mouse was detected five times, and the mean values were calculated after the maximum and minimum values were eliminated.

After mice were sacrificed, hindlimb muscles were collected, and muscle mass of quadriceps was weighed by electronic balance and recorded for analysis.

### Determination of serum estradiol concentration by ELISA

After whole-body vibration training and estradiol supplementation, serum estradiol concentration was determined by Estradiol ELISA kit (Beyotime Biotechnology, Shanghai, China) according to manufacturer's instructions, and the OD value was detected at 450 nm.

### Western Blotting

After exercise or estradiol intervention, hindlimb muscle tissues were harvested, and total protein was extracted using RIPA lysis buffer (Beyotime Biotechnology, Shanghai, China) containing protease and phosphatase inhibitor cocktail (Beyotime Biotechnology, Shanghai, China). Then the protein concentration was measured by enhanced BCA protein assay kit (Beyotime Biotechnology, Shanghai, China). ~ 50 μg protein were separated on SDS-PAGE gel and transferred to the PVDF membranes (Millipore, Ireland, USA). After blocking with 5% (w/v) fat-free milk at room temperature (RT) for 2 h, the membranes were incubated with primary antibodies at 4 ℃ overnight: p-Akt (Ser473) (4060S, 1:1000), Akt (4685S, 1:1000), p-mTOR (Ser2448) (2971S, 1:1000), mTOR (2983S, 1:1000), p-FOXO1 (Thr24) (2599S, 1:1000), FOXO1 (2880S, 1:1000), all primary antibodies were purchased from Cell Signal Technology, Inc. Thereafter, the membranes were incubated with HRP-conjugated anti-mouse/rabbit secondary antibodies at RT for 2 h, and the blots were visualized by ECL reagent (Tanon, China), then detected by automatic chemiluminesence image analysis system (Tannon 5200, Tannon Technology Co., Ltd, China).

### Statistical Analysis

All the data was presented as mean ± SD, and statistical analysis was performed by SPSS 21.0. The difference between two groups was analyzed by Student's t-test, differences among four or five groups were performed by one-way ANOVA and post hoc comparisons using LSD tests, the correction analysis between E2 and p-Akt/Akt, p-mTOR/mTOR or p-FoxO1/FoxO1 was performed by Pearson correlation coefficient and linear regression analysis. p < 0.05 was consider as statistically significant.

## Results

### Establishment of ovariectomized mice model

**Figure 1:**
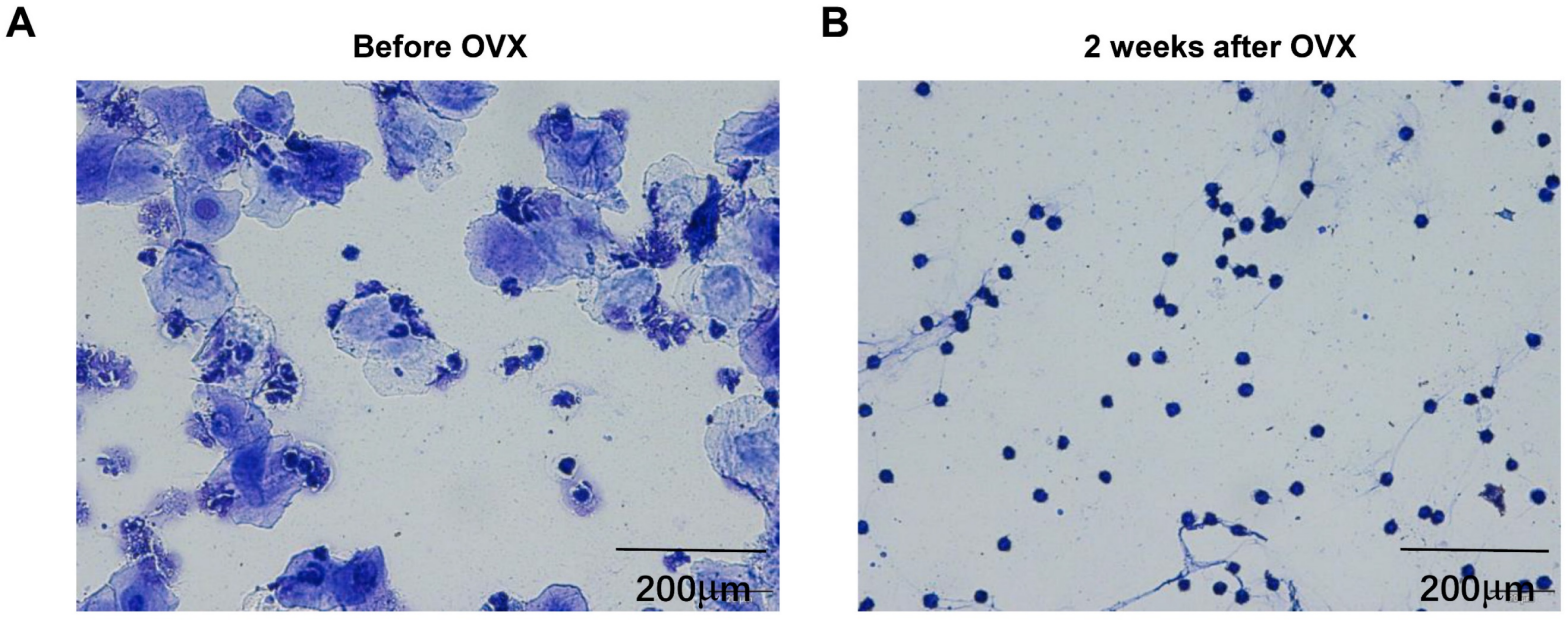
** Vaginal secretions before and after ovariectomy.** The pictures represented the vaginal smear of mice (A) before and (B) 2 weeks after ovariectomy. The magnification was ×200.

To confirm ovariectomy was successfully performed, vaginal secretions were examined before and after ovariectomy. As shown in Figure 1, the vaginal secretions taken from mice before ovariectomy contain a great deal of squamous epithelial cells, whereas comprise many round leukocytes in mice after ovariectomy, suggesting that ovariectomized mice model was successfully established.

### Ovariectomy altered body composition, and reduced muscle mass and strength

**Figure 2:**
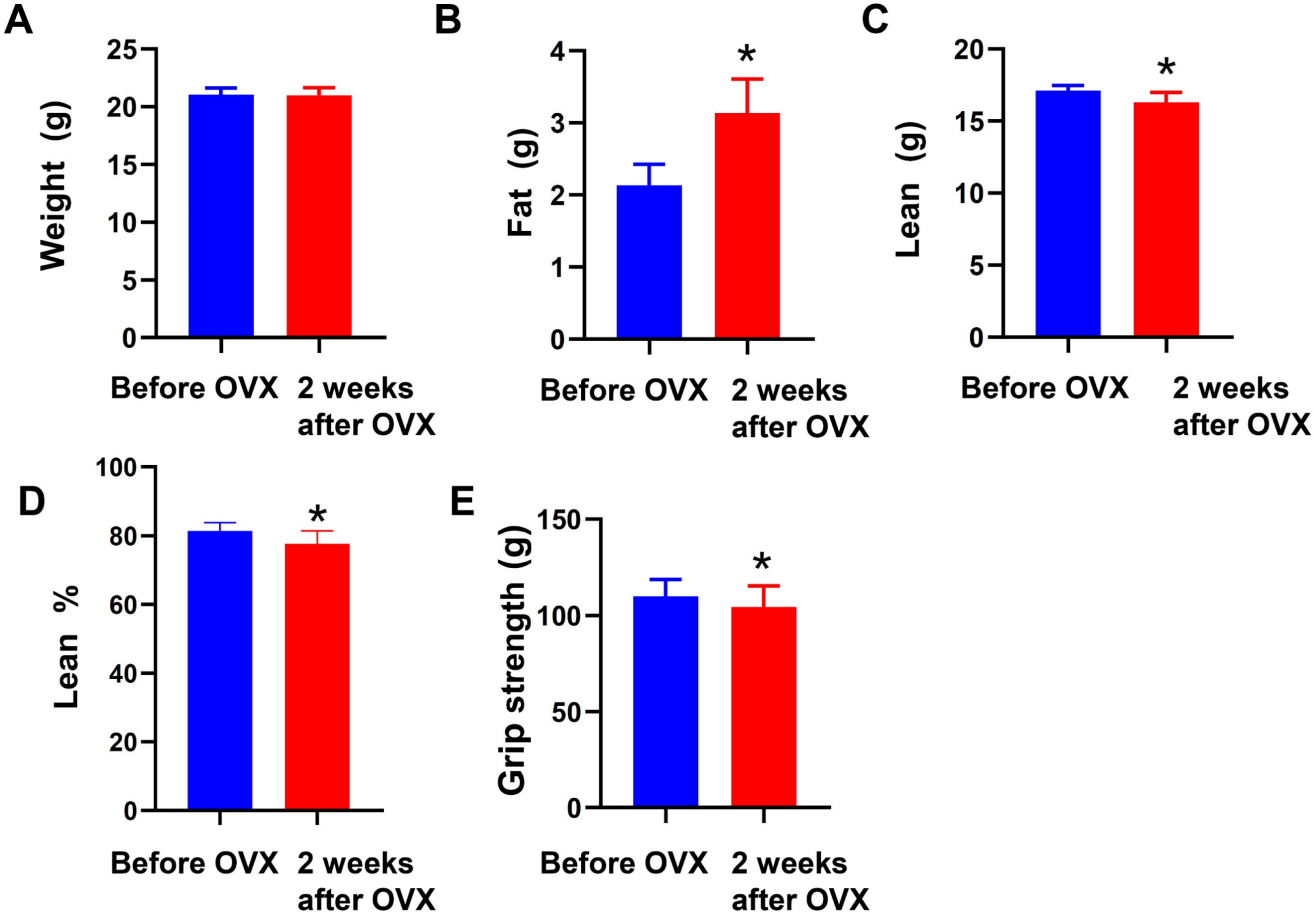
** Ovariectomy altered body composition, and reduced muscle mass and strength in female mice.** (A) Body weight was weighed by electronic scales and (B-D) body composition including fat content, lean body mass and lean percent and (E) muscle strength were determined by Echo MRI-100 body composition analyzer and grip-strength meter before and 2 weeks after ovariectomy, respectively. ^*^ p<0.05 vs Before.

As shown in Figure 2, we found that ovariectomy induced alterations of body composition in female mice, characterized by more fat content, less lean body mass and lean percent (Fig. 2B-D), furthermore, the muscle mass and strength of ovariectomized mice were significantly reduced (Fig. 2E).

### Whole-body vibration training and estradiol supplementation reversed ovariectomy-induced changes of body composition, and increased muscle mass and strength

**Figure 3:**
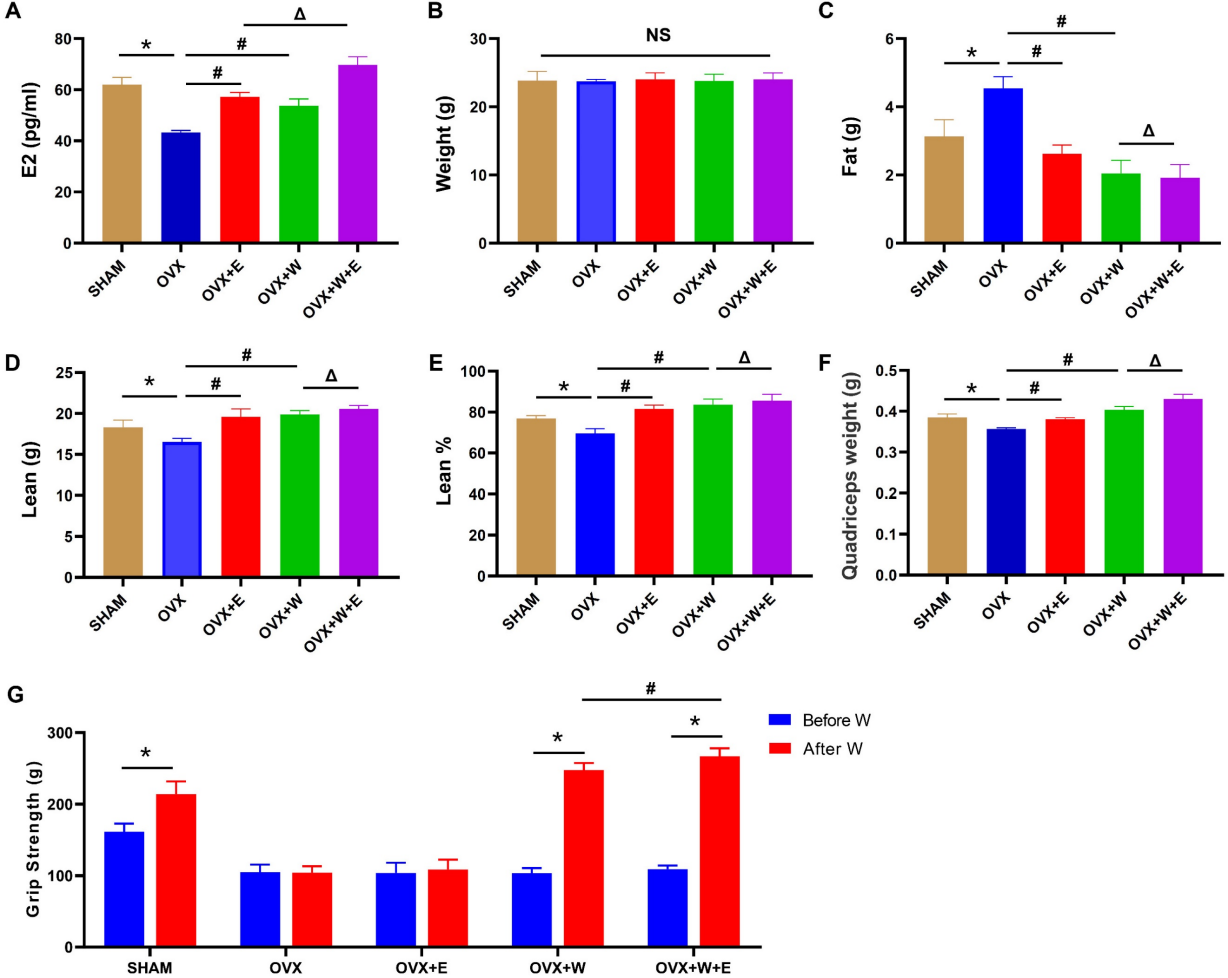
** Whole-body vibration training combined with estradiol supplementation reversed ovariectomy-induced changes in body composition, and increased muscle mass and strength in ovariectomized mice.** After estradiol supplementation and exercise intervention, (A) Serum estradiol concentration was detected by ELISA assay kit. ^*^ p<0.05 vs SHAM group, ^#^ p<0.05 vs OVX group; ^Δ^ p<0.05 vs OVX+E group; (B-F) Body weight, body composition and quadriceps muscle wet weight were determined by electronical scales and Echo MRI-100 body composition analyzer, respectively.^ *^ p<0.05 vs SHAM group, ^#^ p<0.05 vs OVX group; ^Δ^ p<0.05 vs OVX+W group. (G) Grip strength was determined by grip-strength meter.^ *^ p<0.05 vs Before W; ^#^ p<0.05 vs OVX+W group after exercise intervention. SHAM: mice experienced sham operation; OVX: mice undertaken ovariectomy; OVX+E: mice subjected to ovariectomy plus estradiol supplementation; OVX+W: mice experienced ovariectomy plus whole-body vibration training; OVX+W+E: mice exposed ovariectomy plus whole-body vibration training and estradiol supplementation.

Compared with SHAM group, ovariectomy significantly reduced the level of serum estradiol, while WBV and estradiol supplementation remarkably increased serum estradiol level in ovariectomized mice, and the increased degree induced by WBV combined with estradiol supplementation was greater than that by WBV alone (Fig. 3A).

WBV and estradiol supplementation reversed ovariectomy-induced changes of body composition, manifested by reduced fat content (Fig. 3C) and increased lean mass and lean percent in ovariectomized mice (Fig. 3D and E), although had no effect on body weight (Fig. 3B). Additionally, WBV and estradiol supplementation also remarkably increased quadriceps muscle mass (Fig. 3F), but only whole-body vibration training significantly increased grip strength in OVX mice (Fig. 3G). Furthermore, the altered degrees of WBV combined with estradiol supplementation on body composition (including fat content and lean mass) and muscle mass and grip strength were greater than that of WBV or estradiol supplementation alone.

### Whole-body vibration training and estradiol supplementation increased muscle mass and strength of ovariectomized mice via Akt-mediated mTOR and FoxO1 signal pathways

**Figure 4:**
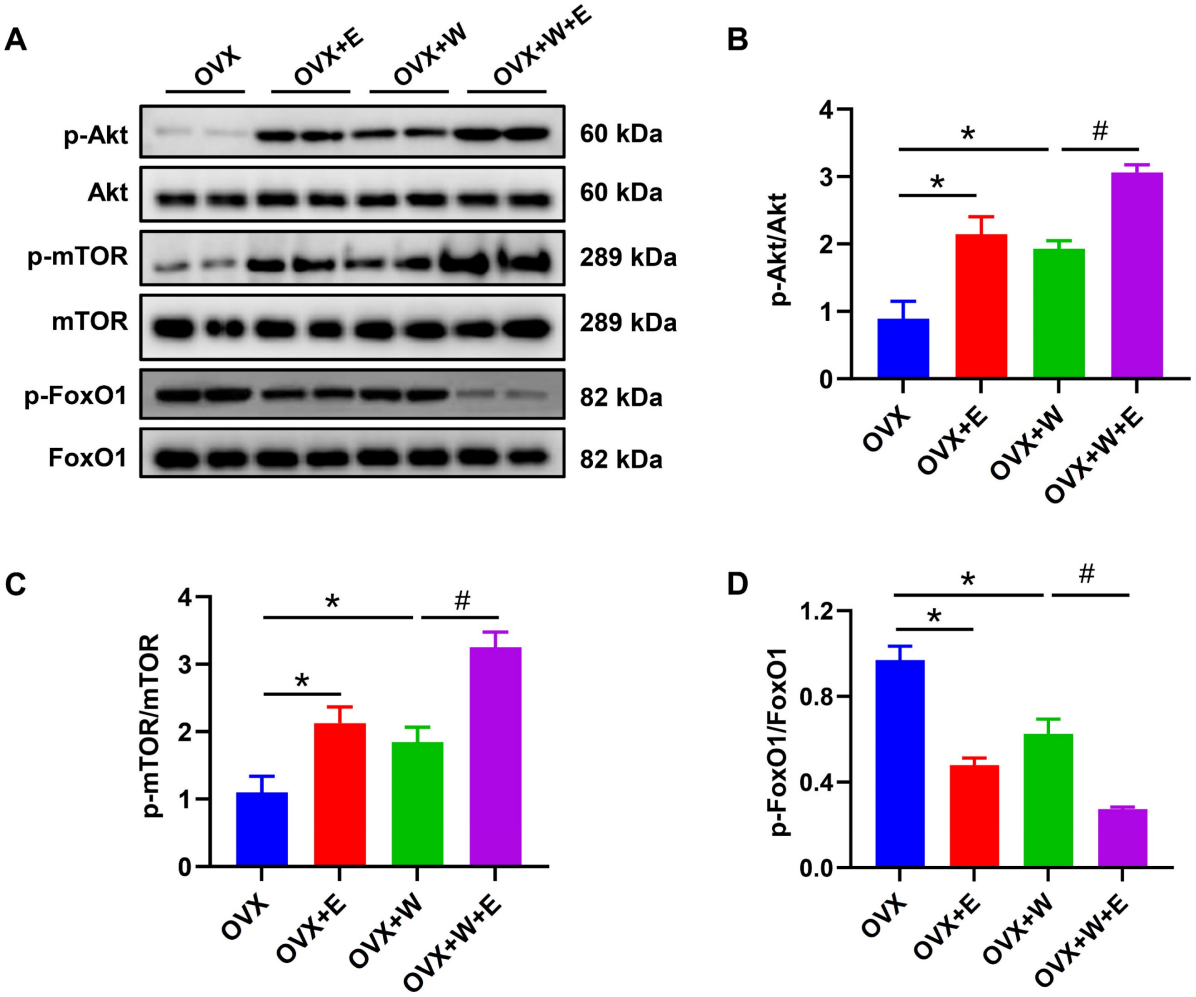
** Whole-body vibration training combined with estradiol supplementation increased muscle mass and strength of ovariectomized mice via Akt-mediated mTOR and FoxO1 signal pathways.** After estradiol supplementation and exercise intervention finished, the total and phosphorylated levels of Akt, mTOR and FOXO1 were detected by western blotting. (A) The presentive bands of p-Akt, Akt, p-mTOR, mTOR, p-FoxO1 and FoxO1 protein, (B) the activities of Akt, mTOR and FOXO1 calculated by p-Akt/Akt, p-mTOR/mTOR, and p-FoxO1/FoxO1, respectively. ^*^ p<0.05 vs OVX group; ^#^ p<0.05 vs OVX+E group.

Compared with OVX mice, WBV and estradiol supplementation alone significantly enhanced AKT and mTOR activities (Fig.4B and C), but reduced the activity of FoxO1 (Fig.4D) in plantar muscle, furthermore, the altered degrees of WBV combined estradiol supplementation on Akt, mTOR and FOXO1 activities were greater than that by WBV or estradiol supplementation alone, which suggested that the effects of WBV and estradiol supplementation on muscle mass and strength may be related to Akt-mediated mTOR and FoxO1 signal pathways.

### Serum estradiol concentration was positively related with the ratios of p-Akt/Akt and p-mTOR/mTOR, but inversely correlated with p-FoxO1/FoxO1

**Figure 5:**
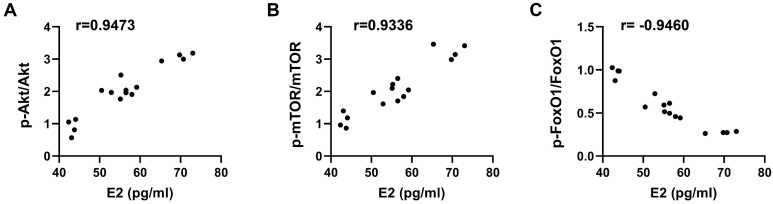
** Serum estradiol concentration was positively related with the levels of p-Akt/Akt and p-mTOR/mTOR, and inversely correlated with p-FOXO1/FOXO1.** Pearson correlation coefficient between estradiol and ratios of p-Akt/Akt (A), p-mTOR/mTOR (B), and p-FoxO1/FoxO1 (C), respectively was analyzed.

We analyzed the correlation between estradiol and ratios of p-Akt/Akt, p-mTOR/mTOR, and p-FoxO1/FoxO1 by Pearson correlation coefficient, and found that serum estradiol concentration was positively related with the ratios of p-Akt/Akt (r=0.9473, p<0.05) and p-mTOR/mTOR (r=0.9336, p<0.05) (Fig. 5A and B), while inversely correlated with p-FoxO1/FoxO1 (r=0.9460, p<0.05) (Fig. 5C), which indicated that the improvements of WBV or estradiol supplementation on muscle mass and strength may be achieved through elevating serum estradiol level, and activating Akt and mTOR while inhibiting of FoxO1 pathways.

## Discussion

### Successful establishment of ovariectomized mice

The ovarian failure in menopause women has been linked to the reduction of lean body mass and increase of fat content, that is different from alterations elicited by aging. For age-related sarcopenia, sedentary lifestyle is attributed to be the possible causative factors, whereas menopause-related sarcopenia is owing to reduced estrogen. OVX is commonly used in mice to mimic menopause condition in women. In female mature mice, the vaginal secretion contains large number of squamous epithelial cells, while in ovariectomized mice, because the disappearance of ovary function and the decline of estrogen secretion, the vaginal secretion is dominated by round leukocyte. In the present study, we also found similar phenomenon that the vagina of mice before experiencing ovariectomy contained a large number of squamous epithelial cells, whereas comprised many round leukocytes after removing ovaries, which suggested the successful establishment of ovariectomized mice.

### Whole-body vibration training and estradiol supplementation reversed ovariectomy-induced alterations in body composition and increased muscle mass and strength

A decrease in estrogen has been demonstrated to cause muscle mass loss and the decline of muscle strength in menopause women 29. Exercise has been well-established to be an effective approach to increase muscle mass and strength in young and elderly individuals. Studies from human and animals indicated that exercise training, especially resistance exercise, could effectively prevent sarcopenia in older individuals, but a considerable proportion of elderly people are unwilling to comply with resistance training program, as its beneficial effects are dependent on high loads 9. Whole-body vibration has been proposed to be a prospective strategy to counteract the decrements of muscle mass and strength in older people with sarcopenia. Although growing evidence indicated that WBV significantly increased muscle strength and physical performance in older adults with sarcopenia 12, 30, there is no whole-body vibration training program to improve muscle mass and strength in ovariectomized mice. In the present study, we found that whole-body vibration training with 30 Hz, 5 d/week for 8 weeks remarkably increased muscle strength of ovariectomized mice, and altered body composition characterized with less fat and more lean body mass (including total lean body mass and lean mass percent). To our knowledge, this is the first report that established a whole-body vibration training program to increase muscle mass and strength of ovariectomized mice, which is beneficial to deeply study the mechanism about the effects of WBV on muscle mass and strength. Furthermore, we also found that the beneficial effects of WBV on muscle mass and strength were influenced by estrogen level, because the improvements of WBV combined with 17β-estradiol on muscle strength and body composition were greater than WBV alone.

### Whole-body vibration training and estradiol supplementation improved ovariectomy-induced decrement of muscle mass and strength via increasing serum estradiol and regulating Akt-mediated mTOR and FoxO1 pathways

The increase of protein synthesis and decrease of protein degradation are crucial for exercise-induced muscle hypertrophy and muscle mass gain 31. Akt-mediated mTOR and FoxO1 pathways are important regulators of protein synthesis and degradation, respectively. A great body of evidence from *in vivo* and *in vitro* studies demonstrated that upregulation of Akt-mTOR and downregulation of FoxO1 were attributed to muscle atrophy 25, 32, while increasing Akt-mTOR and/or suppressing FoxO1 signaling pathway promotes muscle hypertrophy 23, 33. Resistance and aerobic exercise 34, 35 as well as electrical pulse stimulation *in vitro* 36 promoted muscle hypertrophy via activating Akt-mTOR signal pathway; the prevention of skeletal muscle atrophy in ovariectomized rats by weight-bearing exercise was also achieved through activating Akt-mTOR and suppressing FoxO1 pathways 37.

Besides ovary, skeletal muscle is another source synthesizing and secreting estradiol. Our previous study demonstrated that passive stretching of muscle cells *in vitro* induced a remarkable increase of estradiol and promoted glucose uptake 38. In the present study, we demonstrated that WBV significantly increased serum estradiol level, which may be attributed to the increase of estradiol synthesis and secretion elicited by WBV-induced muscle contraction. Estradiol participated in the regulation of glucose metabolism through Akt-mTOR and FoxO1 activity, for example, estradiol promotes glucose uptake and insulin secretion of islet β cells via Akt-mTOR signal pathway 27, improves insulin sensitivity and suppresses gluconeogenesis in ovariectomized mice via hepatic FoxO1 28. We found that WBV increased Akt and mTOR activities and decreased the activity of FoxO1, accompanied with upregulation of serum estradiol, moreover, the serum estradiol level was positively related to Akt and mTOR activity, while inversely correlated with FoxO1 activity, which suggested that WBV might increase muscle mass and strength via activating Akt-mTOR and suppressing FoxO1 signaling pathway. In addition, it should be noted that the enhanced degrees of Akt and mTOR activities and inhibitory degree of FoxO1 activity induced by WBV combined with estradiol supplementation were higher than WBV alone, which suggested the improvements of WBV on muscle mass and strength may be influenced by estrogen status.

## Conclusion

In summary, whole-body vibration training could significantly improve muscle mass and strength in ovariectomized mice via activating Akt-mTOR and suppressing FoxO1 signal pathway, and the improvements of muscle mass and strength by whole-body vibration training was influenced by estrogen level.
